# Constraint-Induced Movement Therapy Promotes Contralesional Red Nucleus Plasticity and Increases Bilateral Motor Cortex-to-Red Nucleus Projections After a Large-Area Stroke

**DOI:** 10.1155/bn/3631524

**Published:** 2025-03-23

**Authors:** Peile Liu, Jian Hu, Beiyao Gao, Yan Hua, Ying Xing, Yulong Bai, Nan Liu

**Affiliations:** ^1^Department of Rehabilitation Medicine, Fujian Medical University Union Hospital, Fuzhou, China; ^2^Department of Rehabilitation Medicine, Huashan Hospital, Fudan University, Shanghai, China; ^3^Department of Rehabilitation Medicine, China-Japan Friendship Hospital, Beijing, China

**Keywords:** constraint-induced movement therapy, ischemic stroke, motor function, neural rehabilitation, red nucleus

## Abstract

For decades, scientists have explored the patterns of neural network remodeling that occur after a stroke. Several studies have shown that both motor cortexes (MCs) undergo crucial remodeling after cerebral ischemia. However, the mechanism by which corticofugal fibers are remodeled is not well understood. Therefore, this study was aimed at investigating the changes in the bilateral red nucleus (RN) and MC–RN projections during recovery from a large-area stroke in a rat stroke model with or without constraint-induced movement therapy (CIMT). A large-area middle cerebral artery occlusion (MCAO) model was established in rats using the Longa method. CIMT was initiated 7 days after MCAO and continued for 1, 2, or 3 weeks. Rats in the control group underwent spontaneous recovery. Locomotor impairment was evaluated using the CatWalk automated gait analysis system, and overall neurological function was evaluated with the modified neurological severity score. Bilateral MC–RN projections were visualized by labeling fiber tracts with an anterograde tracer. Postsynaptic density 95 (PSD95), growth-associated protein 43 (GAP43), and synaptophysin expression levels in the RN were detected using western blotting and immunohistochemistry. The results showed that CIMT promoted motor recovery after a stroke, increased levels of GAP43 and PSD95 in the contralesional but not ipsilesional RN, and increased projections from the MC to the bilateral RN. Thus, CIMT promotes neuroplasticity after a large-area stroke by stimulating axon outgrowth, improving postsynaptic membrane function in the contralesional RN, and increasing bilateral projections of the MC–RN. These results provide evidence for the therapeutic efficacy of CIMT in restoring motor function and help with understanding RN plasticity after a large-area stroke.

## 1. Introduction

Strokes cause long-term damage to brain structures and physical function. Despite substantial functional recovery that can occur during the initial weeks following a stroke, more than 50% of stroke survivors exhibit hemiparesis at 6 months, which is especially significant because motor recovery plateaus after 3–6 months [1]. Constraint-induced movement therapy (CIMT), which involves the repeatedly forced use of a more severely impaired limb, was designed to counteract the learned nonuse of a paralyzed limb that occurs after a stroke. Several high-quality studies have demonstrated that poststroke CIMT can promote functional recovery [2, 3], although the mechanism underlying this effect is poorly understood.

Scientists have spent decades exploring the patterns of neural network remodeling that can occur after a stroke. Due to the presence of diaschisis, not only the peri-infarct area but also more distant areas, such as the contralesional hemisphere, subcortical area, and area of the brain stem, are involved in disability and recovery after a stroke [4–6]. Numerous studies have demonstrated that both motor cortexes (MCs) undergo vital remodeling after cerebral ischemia [7–9]. An imbalance in interhemispheric inhibition (IHI) is considered a hindrance to poststroke recovery. After a stroke, inhibition of the contralateral MC to the ipsilesional MC is believed to increase, causing interference with voluntary movement of the paretic limb [10]. However, the role that IHI plays in poststroke motor recovery remains unclear. Recent research suggested that abnormal IHI is not causally associated with poststroke motor impairment but rather related to better recovery [11, 12]. The relationship between the bilateral cortex and poststroke motor recovery is complex. Sato et al. [13] showed that after a stroke occurs in a large cortical area, the contralesional motor and sensory cortex axons cross the midline and sprout on the denervated side of the cervical spinal cord. In contrast, axons of the spared ipsilesional motor area grow on the denervated side after a small stroke [13]. In rats with a small unilateral internal capsule hemorrhage, changes in the ipsilesional corticorubral pathway were found to contribute to functional recovery induced by forced limb use [14]. However, in large-area strokes, reorganization of the contralesional hemisphere may be more important than that of the ipsilesional hemisphere [8].

In addition to the cortex, the mechanism by which corticofugal fibers are remodeled is not well understood. We have previously reported that the MC and red nucleus (RN) are involved in CIMT-induced neural network reorganization [15]. The RN, located in the rostral midbrain, is related to grasping, motor control, and coordination [16] and is the origin of the rubrospinal tract (RST). In the spinal cord, the RST and corticospinal tract (CST) terminate in proximity and are functionally related, suggesting that the RN may be able to compensate for CST injury after a stroke. In humans, extensive input from the cerebral cortex to the RN exists. After adult macaque monkeys experience primary MC lesions, a strong decrease in the density of corticorubral motor projections occurs, suggesting that direct cortical influence on the RN is reduced [17]. However, the roles that the bilateral RN and MC–RN projections play during recovery from large-area strokes have not been comprehensively studied.

**Figure 1:**
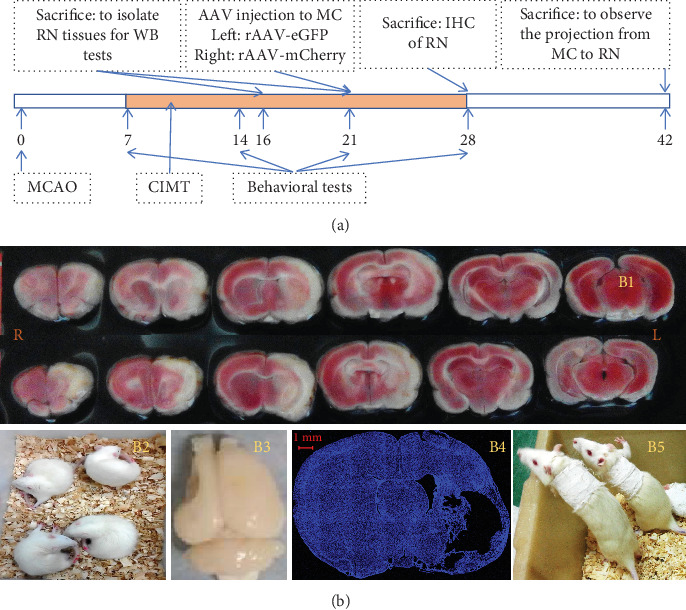
(a) Experimental scheme of this study. (b) MCAO model establishment and CIMT. (b1) TTC staining the day after MCAO. (b2) Circling behavior of rats the day after MCAO. (b3, b4) Large area of liquefactive necrosis observed on D21 after MCAO. (b5) Forced use of the impaired forelimb in the CIMT group.

In the present study, we compared the expression levels of axon growth, presynaptic plasticity, and postsynaptic plasticity markers in the RN of rats treated with CIMT and controls and examined differences in bilateral projections of the MC–RN using an anterograde tracer in a rat large-area middle cerebral artery occlusion (MCAO) stroke model. See Figure 1a for the experimental scheme.

## 2. Materials and Methods

### 2.1. MCAO Model

Adult male Sprague–Dawley rats were housed at a constant temperature (25°C) and humidity (55% ± 5%) on a 12:12 h light:dark cycle with free access to food and water. The experimental procedures involving rats were carried out in accordance with the protocols approved by the Animal Welfare and Ethics Group, Department of Laboratory Animal Science, Fudan University (Ethics Approval Number: 2020 Huashan Hospital JS-152). All outcome assessments were performed by investigators blinded to the treatment.

A large-area stroke MCAO model [8] was established using the Longa method in rats weighing 250–280 g. Briefly, after anesthetization with 2% sodium pentobarbital (40 mg/kg), the left common carotid artery, internal carotid artery (ICA), and external carotid artery (ECA) were exposed. A nylon thread coated with silica gel at one end (Cat. No. 2636A5; Beijing Cinontech Co., Beijing, China) was inserted into the ICA through a small hole made in the ECA. The nylon thread was advanced 19–20 mm into the ICA to occlude the MCA and kept in place for 1.5 h before being removed to allow reperfusion. Laser Doppler flowmetry was used to monitor blood flow in the area of the brain supplied by the left MCA during the operation (1 mm posterior and 5 mm lateral to the bregma). A sharp decrease in blood flow to 30% of the baseline value after insertion of the nylon thread and an increase to > 50% of the baseline value after its removal were considered evidence of successful ischemia and reperfusion. On the day after MCAO, 2,3,5-triphenyltetrazolium chloride (TTC) staining was performed on two rats to determine the location and size of the infarction (Figure 1B1). Briefly, rats were decapitated after deep anesthesia, and their brains were then removed and cut into 1-mm-thick slices that were thereafter immersed in TTC solution for 30 min at 37°C. The slices were photographed with a Xiaomi Redmi 6 device for further observation.

Neurological function was scored on a 5-point scale (0–4) on the day after MCAO (0, *no apparent deficits*; 1, *failure to fully extend the forepaw on the contralesional side*; 2, *circling to the contralesional side* (Figure 1B2); 3, *falling to the contralesional side*; and 4, *unable to walk spontaneously and exhibiting a reduced level of consciousness*). Rats with a score of 1, 2, or 3 after MCAO surgery were used for the experiments and randomly divided into CIMT and control groups.

### 2.2. CIMT

On the seventh day after surgery (D7), the intact arm (left) of rats in the CIMT group was fixed onto the chest with plaster for 1, 2, or 3 weeks to force the use of the affected arm (right) (Figure 1B5). The plasters were released for 3 h a day, during which no food or water was available. The rats in the control group underwent spontaneous recovery.

### 2.3. Behavioral Tests

Locomotor impairment was evaluated using the CatWalk automated gait analysis system (Software Version XT 10.0; Noldus, Wageningen, the Netherlands) [18, 19]. The camera and walkway area were set according to the manufacturer's recommendations. The data selection parameters were as follows: minimum number of consecutive steps per run = 10; average speed range = 20‐90 cm/s; and maximum allowable speed variation = 80%. No food or water restrictions were imposed during the experiments; however, a few food pellets were placed in the goal box as a motivating cue for runs. Evaluation was performed at the same time (between 10 am and 5 pm) on D7, D14, D21, and D28 after MCAO. The plastic cast was removed on the day prior to the test. Each trial comprised three runs. Each footprint was automatically labeled, manually inspected, and adjusted, whereafter gait parameters were automatically generated. We focused on the following parameters: temporal paw (initial dual stance, terminal dual stance, and stand duration), comparative paw (duty cycle), kinetic paw (body speed), and interpaw coordination (phase dispersion: diagonal and ipsilateral paw pairs) parameters. Decreases in the initial dual stance, terminal dual stance, stand duration, and duty cycle and an increase in body speed would indicate an improvement in locomotor function [20]. Normal values for the diagonal and ipsilateral pairs of paws were 0% and 50%, respectively.

Overall neurological function was assessed using the modified neurological severity score (mNSS), which comprehensively evaluates neurological deficits associated with movement, sensation, balance, and reflexes, with a total score of 18 points, where higher scores indicate a more serious deficit [21].

### 2.4. Anterograde Tracing

Anterograde tracing was performed using adeno-associated virus (AAV) vectors to label bilateral projections from the MC–RN. Briefly, on D22–D25 after MCAO, the rats were anesthetized and immobilized in a stereotaxic apparatus. AAV vectors expressing enhanced green fluorescent protein (eGFP) or mCherry were delivered via a microliter Hamilton syringe at a flow rate of 80 nL/min controlled by an electrical pump. Two injections were performed on each side of the MC forelimb area (2.5 mm anterior–posterior (AP), 2 mm medial–lateral (ML), and 1.5 mm dorsal–ventral (DV); 2.5 mm AP, 3 mm ML, and 1.5 mm DV). The left cortex was injected with rAAV-EF1a-eGFP-WPRE-PA, and the right cortex with rAAV-EF1a-mCherry-WPRE-hGH-pA (0.4 *μ*L/site). The syringe was kept in place for 5 min before and 10 min after the injection to prevent leakage. Three weeks later, rats were transcardially perfused with 0.1 M phosphate-buffered saline and 4% paraformaldehyde, and the brain was then dissected, postfixed, cryoprotected, and cut into sections at a thickness of 30 *μ*m on a cryostat microtome. Two to four sections per brain were photographed at the RN level under a fluorescence microscope, and bouton-like swelling (varicosities) positive for eGFP or mCherry in the RN and cerebral peduncle (CP) counted at a 400× magnification. To eliminate interindividual differences, the ratio of varicosities in the RN to those in the CP (RN/CP) was calculated to allow comparisons between groups [14].

### 2.5. Western Blotting

On D16 and D21, bilateral RNs were isolated for western blotting. Brain tissue was homogenized and centrifuged at 4°C and 14,000 rpm for 30 min. Proteins were separated via electrophoresis on a sodium dodecyl sulfate-polyacrylamide gel at low voltage and transferred to a polyvinylidene membrane that was blocked with 5% skim milk dissolved in Tris-buffered saline with 0.1% Tween-20 at room temperature (20°C~25°C) for 2 h. The membranes were then incubated with primary antibodies against growth-associated protein 43 (GAP43) (1:3000, No. ab75810; brain tissue from D21; Abcam, Cambridge, United Kingdom), synaptophysin (1:8000, Cat. No. ab32127; brain tissue from D16; Abcam), postsynaptic density 95 (PSD95) (1:1000, Cat. No. GB11277; brain tissue from D16; ServiceBio, Wuhan, China), and actin (1:1000, Cat. No. GB12001; ServiceBio). The secondary antibody used was horseradish peroxidase-labeled goat anti-rabbit IgG (1:2000, Cat. No. A0208; Beyotime, Shanghai, China). Protein bands were enhanced using a chemiluminescence system (ECL kit; Millipore, Burlington, MA, United States), and band images were analyzed with the Bio-Rad ChemiDoc XRS system (Bio-Rad, Hercules, CA, United States).

### 2.6. Immunohistochemistry

On D28, transcardial perfusion was performed with a saline solution followed by paraformaldehyde. The brain was isolated for detection of GAP43 expression using immunohistochemistry. Three brain slices (RN level, 20 *μ*m thick) from each sample were prepared with sodium citrate buffer (0.01 M, pH 6.0) for 30 min at 80°C, blocked with 10% donkey serum for 2 h at room temperature, and incubated with anti-GAP43 antibody overnight at 4°C. The slices were then incubated with a donkey anti-rabbit IgG secondary antibody (Alexa Fluor 594, 1:400; 34212ES60; Yeasen, Shanghai, China) for 2 h at room temperature. Finally, the slices were mounted using an antifluorescence quencher with 4⁣′,6-diamidino-2-phenylindole. Six visual fields of the immunolabeled RN (three each on the left and right sides) in each brain section were photographed using a confocal microscope (FV3000; Olympus, Tokyo, Japan) at 400× magnification. The GAP43 puncta density was counted using the “Count” tool in Image-Pro Plus 6.0 (Media Cybernetics, Rockville, MD, United States).

### 2.7. Statistical Analysis

Statistical analyses were performed using SPSS v18.0 (IBM Corp., Armonk, NY, United States). A two-way analysis of variance was used to analyze data obtained from the CatWalk test and mNSS. All other data were evaluated for normality; an unpaired (or adjusted) *t*-test and nonparametric Kruskal–Wallis test were used for normally and non-normally distributed data, respectively. Data are presented as the mean ± SEM. All tests were two-tailed, and the significance level was set at *p* < 0.05.

## 3. Results

### 3.1. CIMT Promotes Motor Recovery After MCAO

**Figure 2:**
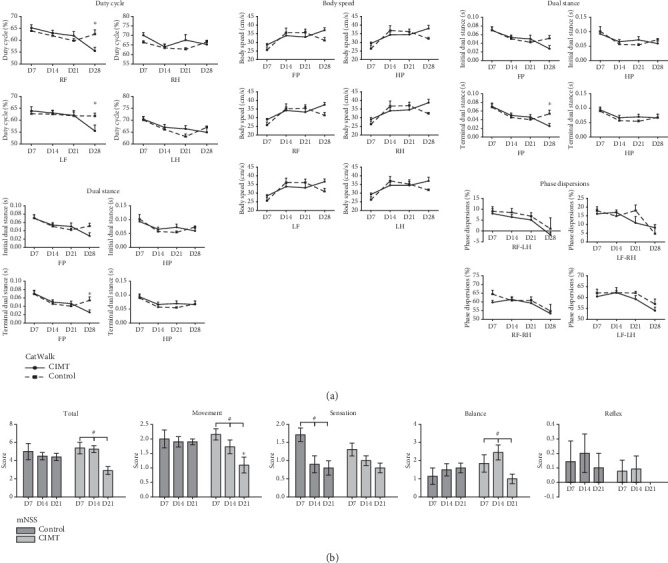
CIMT promotes motor recovery in rats after a stroke. (a) Gait analysis using the CatWalk test. CIMT group: D7, *n* = 7; D14, *n* = 7; D21, *n* = 4; D28, *n* = 4. Control group: D7, *n* = 6; D14, *n* = 5; D21, *n* = 5; D28, *n* = 3. Abbreviations: RF, right front paw; RH, right hind paw; LF, left front paw; LH, left hind paw; FP, front paws; HP, hind paws. (b) Assessment of neurological function based on the mNSS. CIMT group: D7, *n* = 13; D14, *n* = 11; D21, *n* = 10. Control group: D7, *n* = 7; D14, *n* = 10; D21, *n* = 10. ⁣^∗^*p* < 0.05, CIMT versus control group at the indicated time point; ^#^*p* < 0.05, significant difference between indicated time points within a group.

In the gait analysis after 3 weeks of training, the stand duration, terminal dual stance, and duty cycle of the forepaws were significantly shorter (indicating better motor function) in the CIMT group than in the control group (stand duration of the right front paw: 0.224 ± 0.012 s vs. 0.292 ± 0.022 s, *p* = 0.037; stand duration of the left front paw: 0.221 ± 0.016 s vs. 0.301 ± 0.028 s, *p* = 0.016; terminal dual stance of the front paws: 0.025 ± 0.006 s vs. 0.054 ± 0.008 s, *p* = 0.020; duty cycle of the right front paw: 55.484% ± 1.526% vs. 62.541% ± 1.770%, *p* = 0.003; duty cycle of the left front paw: 55.543% ± 2.428% vs. 61.803% ± 1.101%, *p* = 0.031). Both groups showed improvement in interpaw coordination parameters over time, with no significant difference between groups (Figure 2a).

For neurological function evaluated using the mNSS, no significant differences between time points in the total score and balance and movement experiment scores were observed in the control group (total score: *F*[2, 55] = 0.280, *p* = 0.757; balance experiment score: *F*[2, 55] = 0.290, *p* = 0.749; movement experiment score: *F*[2, 55] = 0.54, *p* = 0.988). However, in the CIMT group, significant differences were observed over time in the total, balance, and movement experiment scores (total score: *F*[2, 55] = 7.239, *p* = 0.002; balance experiment score: *F*[2, 55] = 3.555, *p* = 0.035; movement experiment score: *F*[2, 55] = 6.554, *p* = 0.003). Sensory experiment scores differed between time points in the control group (*F*[2, 55]) = 5.896, *p* = 0.005) but not in the CIMT group (*F*[2, 55] = 2.275, *p* = 0.121). On D21 after MCAO, the movement experiment score of the CIMT group was significantly lower than that of the control group (1.100 ± 0.876 vs. 1.900 ± 0.316, *p* = 0.013) (Figure 2b).

### 3.2. GAP43 and PSD95 Levels in the RN Are Altered by CIMT

**Figure 3:**
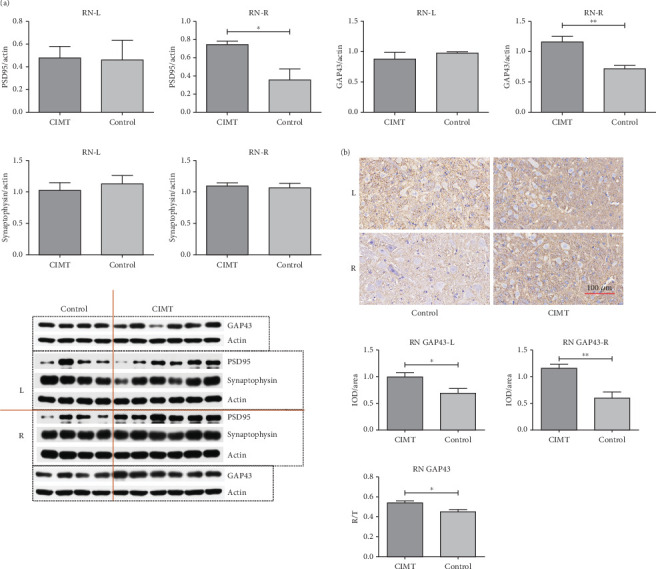
Effects that CIMT has on synaptic protein levels (PSD95, GAP43, and synaptophysin) in the RN. (a) Increased levels of GAP43 and PSD95 in the right RN, but not in the left RN, were induced by CIMT, as determined via western blotting. CIMT group: *n* = 6; control group: *n* = 4. (b) Increase in GAP43 levels observed in the bilateral RN, with a greater increase in the right RN (higher right/total) in the CIMT group, as determined via immunohistochemistry (*n* = 3/group). ⁣^∗^*p* < 0.05; ⁣^∗∗^*p* < 0.01.

Western blotting analysis revealed that GAP43 expression was upregulated in the contralesional RN of the CIMT group compared with that in the control group (1.160 ± 0.093 vs. 0.715 ± 0.059, *t* = 3.554, *p* = 0.008), whereas no significant differences were observed between the two groups in the ipsilesional RN (0.879 ± 0.114 vs. 0.978 ± 0.020, *t* = 0.692, *p* = 0.509) (Figure 3a). However, higher levels of GAP43 were detected in the bilateral RN of the CIMT group compared with those in the control group, as measured via immunohistochemistry (ipsilesional side: 0.993 ± 0.083 vs. 0.688 ± 0.091, *t* = 2.395, *p* = 0.032; contralesional side: 1.157 ± 0.076 vs. 0.596 ± 0.117, *t* = 4.239, *p* = 0.001) (Figure 3b), with contralesional RN accounting for a greater proportion of the total bilateral optical density in the CIMT group compared with that in the control group (right/total, 0.540 ± 0.021 vs. 0.449 ± 0.024, *t* = 2.850, *p* = 0.014). GAP43 is a marker of axonal sprouting. These results suggest that CIMT promotes axonal growth of the contralesional RN on D21 and the bilateral RN on D28.

PSD95 expression was found to be upregulated in the contralesional RN of the CIMT group compared with that in the control group (0.7443 ± 0.0368 vs. 0.357 ± 0.121, *p* = 0.007), as measured via western blotting, whereas no significant differences were observed between the groups in the ipsilesional RN (0.479 ± 0.102 vs. 0.461 ± 0.172, *p* = 0.926) (Figure 3a). The expression of synaptophysin in bilateral RN did not significantly differ between the two groups (right RN, 1.098 ± 0.048 vs. 1.065 ± 0.071, *p* = 0.704; left RN, 1.025 ± 0.120 vs. 1.127 ± 0.134, *p* = 0.594) (Figure 3a). As the most abundant postsynaptic scaffolding protein, PSD95 serves as a marker of postsynaptic plasticity, and as a member of synaptic vesicle proteins, synaptophysin serves as a marker of presynaptic plasticity. These results suggest that CIMT promotes postsynaptic plasticity of the contralesional RN, but not its presynaptic plasticity, on D16.

### 3.3. CIMT Increases Bilateral Projections From the MC–RN

**Figure 4:**
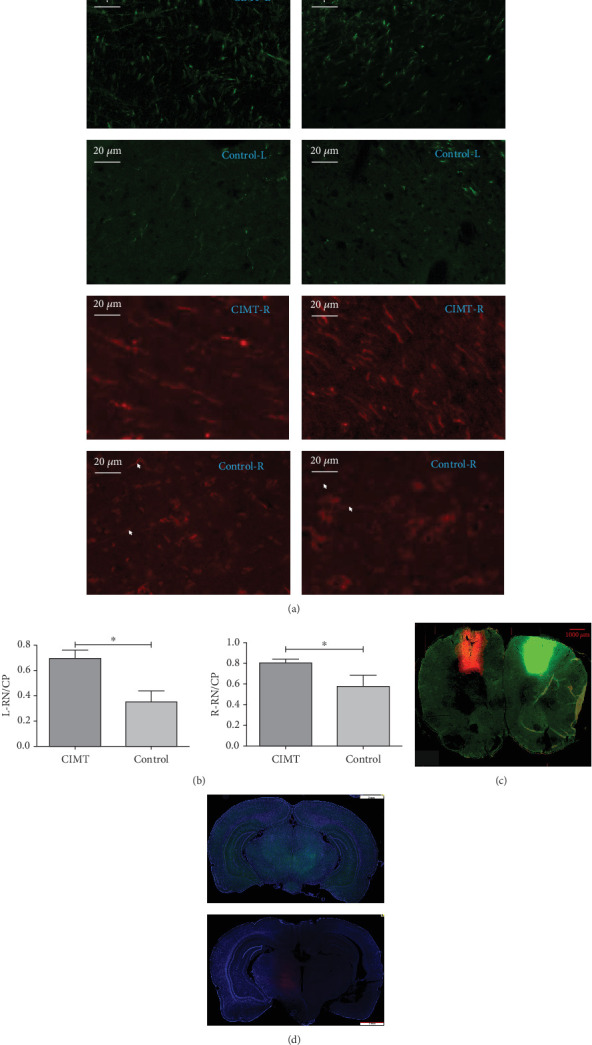
CIMT increases bilateral projections from the MC–RN. (a) Representative fluorescence micrographs of the RN and CP. The arrows point to labeled nerve fibers. (b) Quantitative analysis of the relative fluorescence measured in the RN and CP (*n* = 3/group). The CP was used as a reference to eliminate individual differences in viral infection efficiency. ⁣^∗^*p* < 0.05. (c) Representative fluorescence images of the injection site. (d) Representative images of the ipsilesional and contralesional RN at a lower magnification.

The RN/CP values on the right (mCherry+) and left (GFP+) sides were significantly higher in the CIMT group than those measured in the control group (CIMT vs. control: left RN/CP, 0.697 ± 0.063 vs. 0.350 ± 0.088, *p* = 0.007; right RN/CP, 0.805 ± 0.036 vs. 0.578 ± 0.108, *p* = 0.037) (Figure 4).

## 4. Discussion

Overall, the results showed that rats in the CIMT group exhibited better performance during gait analysis and in their mNSS movement experiment score. However, we did not train the rats for the CatWalk task before surgery. It is thus possible that CIMT improved not only locomotive function but also the ability to learn the task. Owing to the limitations of time, human resources, and equipment resources, we did not conduct any behavioral tests other than the CatWalk test and mNSS. Future research should, therefore, aim to perform more diverse behavioral tests. In fact, our previous study [15] performed a foot-fault experiment, which confirmed that CIMT promotes the recovery of fine movement and grasping in rats.

Reorganization of the RN, a component of the extrapyramidal system, after damage to the CST, has been extensively investigated. The RST has been proposed as a replacement for the CST [22, 23]; the magnocellular portion of the RN is a major component of the RST in lower vertebrates and is well developed in the perinatal and developmental stages in primates [24]. Lawrence and Kuypers showed that after bilateral total pyramidotomy, monkeys regained motor control in a broad range of activities that required independent limb movements, which were likely guided by descending subcorticospinal pathways. Furthermore, these pathways were grouped into the ventromedial and lateral systems, and disruption of the latter resulted in impairments similar to those caused by CST damage [22]. Most fibers in the lateral system are derived from the magnocellular portion of the RN [23]. Furthermore, the number of RST, rubropontine, and rubro–raphe axons increased after bilateral pyramidotomy [25].

Remodeling of the pyramidal and extrapyramidal systems is essential for motor function recovery after a stroke [2, 18, 26]. Rubrofugal fibers have been suggested to be capable of partially replacing the injured CST, and reorganization of the RN after a stroke has been poorly studied, likely due to the small size of this brain structure. This limitation has been partially overcome by the increased spatial resolution of magnetic resonance imaging. A diffusor tensor imaging (DTI) study found that fractional anisotropy (FA) increased and radial diffusivity decreased in the RN on the unaffected side in patients with severely impaired hand function (mean lesion volume: 8.12 cm^3^) compared with those in healthy subjects [27]. Another DTI-based study showed that RN connectivity increased to a greater degree in the unaffected hemisphere than in the affected hemisphere in patients with severe CST injury [28]. Furthermore, in a DTI-based study of patients with relatively small lesions (mean lesion volume: 1.7 cm^3^), changes in the ipsilesional rubrospinal pathway, but not in the contralesional side, were positively correlated with motor function recovery [29]. More recently, another DTI-based study recruited 43 patients with chronic stroke and performed comprehensive motor evaluations and magnetic resonance imaging. Correlation and multiple regression analyses suggested that the microstructural integrity of the ipsilesional RN was positively correlated with the motor function of the affected upper and lower extremities [30].

In the present study, CIMT increased the expression of GAP43 and PSD95, mainly in the contralesional RN after a large-area stroke. However, the immunohistochemical results showed that prolonged CIMT lasting 28 days promoted bilateral RN axon growth. Our study did not find CIMT to exhibit a statistically significant effect on synaptophysin expression in the RN. The possible reasons for this negative result are as follows: (1) the sample size was not sufficiently large, (2) the intervention time was not sufficient to induce significant effects on synaptic vesicles, and (3) CIMT-induced synaptic remodeling had no effect on synaptophysin expression. In conclusion, similar to the previously mentioned DTI studies, our study suggests that the contralesional RN plays a greater role in the recovery of motor function in the paretic forelimb after a large-area stroke.

Cortex fibers mainly target the ipsilateral RN, with very few projecting to the contralateral RN [31]. After unilateral pyramidotomy, loss of the neurite growth inhibitor, IN-1 antigen, was found to result in improved projections from the ipsilesional cortex to the contralesional RN [31]. Additionally, CST neurons innervated the RN after bilateral pyramidotomy [32]. A causal link between increased ipsilesional MC–RN projections and motor function following the formation of internal capsule lesions was demonstrated via anterograde tracing [14]; although this finding was based on very small lesions. A DTI-based study found that compared with normal controls, capsular stroke (mean lesion volume: 0.71 cm^3^) causes decreased FA in the bilateral corticorubral tracts. Furthermore, the FA of the ipsilesional corticorubral tracts and Fugl–Meyer Assessment scores were positively correlated in the capsular stroke group [33]. Consistent with these studies, our findings showed that CIMT increased bilateral projections of the MC–RN after large-area cerebral ischemia.

Our study had some limitations. First, total protein levels in the RN were examined—we did not differentiate between proteins expressed in the RN neurons and those expressed in fibers projected from other areas. Second, we did not establish a causal link between RN reorganization and improved motor function. Third, structural and functional changes in the RN after a stroke and projections between the RN and other areas of the brain were not comprehensively examined. These issues should be addressed in future studies.

## 5. Conclusions

The results of this study indicate that after a large-area stroke, CIMT can promote neuroplasticity by stimulating axon outgrowth, improving the function of the postsynaptic membrane in the contralesional but not the ipsilesional RN, and increasing bilateral projections from the MC–RN. Overall, these results provide evidence for the therapeutic efficacy of CIMT in restoring motor function and improving our understanding of RN plasticity after a large-area stroke.

## Data Availability

The datasets generated for this study can be obtained from the authors upon reasonable request.
